# Narrative Reviews in Contemporary Medical Literature: An Overview of Current Role and Relevance

**DOI:** 10.7759/cureus.110705

**Published:** 2026-06-11

**Authors:** S Bethiun, R C Neil Ananth, Sachu Philip, Janarthanan Shiamala, Thangadurai Maheswaran, Vadivel Ilayaraja, Kaliyamurthy Sivaguru

**Affiliations:** 1 Physiology, Kerala Medical College, Palakkad, IND; 2 Dentistry, C.S.I Mission Hospital, Kanyakumari Medical Mission, Neyyoor, IND; 3 Biochemistry, Al Azhar Medical College and Super Speciality Hospital, Thodupuzha, IND; 4 Prosthodontics, Oral Safe Dental Clinic, Puducherry, IND; 5 Oral and Maxillofacial Pathology, Adhiparasakthi Dental College and Hospital, Melmaruvathur, IND; 6 Oral and Maxillofacial Pathology, Vivekanandha Dental College for Women, Namakkal, IND; 7 Prosthodontist, No. 1 Dental Unit, Assam Rifles, Shillong, IND

**Keywords:** evidence-based medicine, evidence synthesis, knowledge translation, literature review methodology, medical publishing, narrative review, sanra, systematic review

## Abstract

Narrative reviews remain the most prevalent article type in the biomedical literature; however, their methodological properties and clinical relevance are often poorly understood compared with more formal review designs. This review examines the definition and historical context of the narrative review; its role in supporting evidence-based clinical practice; the quality standards and limitations governing its conduct; its relationship with systematic, scoping, and integrative review methodologies; and emerging technological influences, including large language models and machine learning. Evidence from the peer-reviewed literature reveals that narrative reviews serve an indispensable function in synthesizing heterogeneous evidence, communicating complex findings to clinical audiences, and addressing topics for which structured evidence is scarce. However, they are susceptible to selection bias and inadequate reporting and require structured quality appraisal frameworks to maximize their scientific value. Establishing transparent authorship standards and cautiously integrating computational assistance are essential steps in ensuring that narrative reviews remain reliable contributors to medical knowledge.

## Introduction and background

The volume of biomedical literature indexed in international databases has grown at an extraordinary rate over the past four decades, with an estimated 63.4 million hours devoted globally to peer review in the biomedical domain in 2015 alone, and 20% of researchers performing 69-94% of all review work across the field [1]. This proliferation of primary research has simultaneously increased the demand for synthesis and created conditions in which no single clinician can remain current within even a narrow subspecialty, as illustrated by the observation that approximately 25,000 systematic reviews are added to bibliographic databases annually [2,3]. Review articles have long been the primary vehicle for communicating this synthesis to practitioners and policymakers [4]. The narrative review, also known as the traditional or non-systematic review, is the most common type of review article found in peer-reviewed medical journals [4,5]. Its prevalence reflects its adaptability: narrative reviews do not require adherence to the structured methodological frameworks that govern systematic reviews [6] and are therefore capable of addressing topics broadly rather than answering narrow clinical questions [4].

The rise of evidence-based medicine (EBM) from the early 1990s onward subjected all forms of evidence reviews to heightened scrutiny [7,8]. The development of hierarchical evidence frameworks has placed systematic reviews and meta-analyses at the pinnacle of trustworthiness, while narrative reviews have been frequently characterized as susceptible to bias and selective reporting [9,10]. The introduction of reporting tools, such as the Preferred Reporting Items for Systematic Reviews and Meta-Analyses (PRISMA) guidelines for systematic reviews [6] and the Scale for the Assessment of Narrative Review Articles (SANRA) for narrative reviews [4], reflects a growing recognition that all review formats require explicit quality standards. Cabana et al. identified that barriers to guideline adherence among physicians include insufficient awareness and familiarity with the evidence base, gaps that well-conducted narrative reviews are particularly suited to address [11,12]. The growing family of review designs has further expanded the methodological taxonomy available to authors, with scoping and integrative reviews increasingly complementing the narrative and systematic formats [13,14]. Notwithstanding these developments, a consolidated appraisal of the role and relevance of narrative reviews in the contemporary medical evidence landscape remains necessary.

The literature for this review was identified through searches of PubMed using keywords related to narrative reviews. Only articles published in English were included in this review. Priority was given to recent and landmark publications relevant to the scope of this review. This narrative review synthesizes the current evidence regarding the definition, clinical utility, methodological limitations, comparative characteristics, and future trajectory of narrative reviews in the contemporary medical literature.

## Review

Defining the narrative review

Narrative reviews are the most frequently encountered article types in contemporary biomedical publishing. Baethge et al. characterized them as the "staple of medical literature," noting that their prevalence far exceeds that of systematic reviews and randomized controlled trials and that they exercise enormous influence on clinicians in both practice and research [4]. At their core, narrative reviews represent an attempt to synthesize published literature in a manner that is not explicitly systematic; the minimum requirement for the term "systematic" pertains to the method of the literature search but extends, in a broader sense, to a specific research question and a comprehensive account of available studies [4]. This flexibility defines the format and distinguishes it from more constrained review designs.

The taxonomy of literature reviews in medicine has expanded considerably. Smith and Duncan identified that systematic reviews, scoping reviews, and narrative reviews each occupy distinct methodological niches, with traditional narrative reviews remaining valuable particularly when high-quality evidence on a topic is sparse or when population, intervention, comparison, and outcome (PICO)-formatted questions cannot adequately capture the scope of interest [5]. The integrative review, as articulated by Whittemore and Knafl, extends this breadth by permitting the combination of experimental and non-experimental study designs, enabling a more comprehensive account of the knowledge base [14]. Sackett traced the historical trajectory of clinical epidemiology to show that evidence synthesis evolved through five successive revolutions: evidence generation, rapid critical appraisal, efficient storage and retrieval, evidence-based medicine, and synthesis itself [8], and narrative reviews have been a consistent presence throughout each of these transformations, adapting to shifting evidentiary standards while retaining their fundamental synthetic character.

Role of narrative review in clinical practice and evidence synthesis

The clinical utility of narrative reviews extends beyond their educational function. Djulbegovic and Guyatt documented that evidence-based medicine, which emerged in the early 1990s, was initially centered on training clinicians to understand and use published literature and placed the systematic review at the apex of evidence hierarchies [7]. Narrative reviews serve a complementary function by condensing heterogeneous evidence into forms accessible to busy practitioners who lack the time or expertise to directly appraise primary literature. Hayward et al. argued that evaluating the validity of clinical practice guidelines requires clinicians to critically synthesize evidence, a task that expert-led narrative commentary can facilitate by framing technical evidence within a broader clinical context [12].

Barriers to evidence uptake in clinical practice are well documented. Cabana et al. identified seven categories of obstacles preventing physician adherence to practice guidelines, ranging from insufficient awareness and familiarity to self-efficacy deficits and external structural barriers [11]. Narrative reviews are uniquely positioned to reduce the first two categories by consolidating dispersed evidence and providing contextual framing that structured systematic reviews, with their methodological apparatus, cannot readily supply. Chambers et al. found that systematic review producers generate 20 distinct knowledge translation formats (summaries, overviews, and policy briefs) to overcome policymakers' documented difficulty in accessing primary systematic review outputs [15]. Narrative reviews function analogously for clinical end-users, translating the evidence landscape into actionable clinical contexts. This synthesizing role is particularly indispensable in fields where clinical questions are multifaceted, evidence is heterogeneous, or the volume of primary research is insufficient to support a formal meta-analysis.

Quality, limitations, and bias in narrative review

Despite their clinical relevance, narrative reviews are subject to significant methodological limitations that compromise their trustworthiness when not conducted with appropriate rigor. The most fundamental concern is the potential for a selective literature synthesis. Because narrative reviews do not mandate transparent search strategies or pre-specified inclusion criteria, the author's prior beliefs and domain expertise may exert a disproportionate influence over which studies are discussed and how evidence is weighted [10]. Campbell et al. empirically demonstrated this risk in the context of narrative synthesis used in systematic reviews: 95% of public health systematic reviews employing narrative synthesis failed to describe their synthesis methods, and 43% lacked transparent links between the data presented and the conclusions drawn [10].

Drucker et al. enumerated the sources of bias that afflict systematic reviews, including reporting bias from selective data presentation, evidence selection bias driven by publication bias, and the propagation of bias from individual studies included. These vulnerabilities apply with equal or greater force to narrative reviews, which lack protective mechanisms such as pre-specified protocols, systematic search documentation, and formal risk-of-bias assessment [16]. Rosner AL argued that the conventional evidence-based medicine (EBM) pyramid, while placing systematic reviews at its apex, excludes legitimate knowledge domains, including basic science, epidemiology, and health services research, and that even high-quality systematic reviews are susceptible to subjective bias in their inclusion criteria and methodological scoring [9]. Nissen and Wynn observed that clinical case reports-which share characteristics with narrative reviews in their qualitative, experience-driven approach to evidence-are valued for detecting novel phenomena and generating hypotheses, while remaining limited by the absence of generalizability and causal inference capacity [17]. These parallel limitations underscore the need for explicit quality appraisal standards across all non-systematic review formats. The SANRA, with its six-item structure addressing the explanation of importance, aims, literature search, referencing, scientific reasoning, and presentation of relevant endpoint data, represents an important step toward standardizing narrative review quality assessment [4].

Comparison of narrative review with other review modalities

Understanding the distinctive strengths of narrative reviews requires situating them within the broader landscape of review methodologies that have proliferated over the past three decades. The systematic review, as codified by the PRISMA 2020 guidelines, answers three fundamental questions: why the review was conducted, what the authors did, and what they found [6]. With its 27-item reporting checklist, PRISMA 2020 reflects advances in methods for identifying, selecting, appraising, and synthesizing evidence, and its widespread adoption has brought transparency and replicability to the highest tier of the evidence hierarchy [6].

Scoping reviews represent a related but distinct genre of literature. Pham et al. identified 344 scoping reviews published between 1999 and 2012, observing that 74% addressed health topics and that the format was designed to map breadth and depth of knowledge rather than answer narrow efficacy questions-a purpose that partially overlaps with the topical breadth of the narrative review [13]. Formal quality assessment of scoping reviews was performed in fewer than a quarter of cases in that sample, mirroring the under-standardization that characterizes the narrative format [13]. McDonald et al. documented the extraordinary scale of contemporary evidence production, noting that approximately 25,000 systematic reviews are added to databases annually and that registrations in the International Prospective Register of Systematic Reviews, better known as PROSPERO, increased tenfold over five years [2]. This proliferation has created an informational paradox: the expansion of structured review methodology intended to improve evidence quality has simultaneously generated an information overload that narrative reviews, with their synthetic and accessible nature, are well positioned to address. The global peer-review burden, estimated at 63.4 million hours in 2015, with 20% of researchers performing the bulk of all reviews [1], highlights the sustainability challenges that affect all review formats and reinforces the complementary value of the narrative form.

Emerging trends and the future of narrative review

The emergence of large language models (LLMs) presents both opportunities and risks for the production of narrative reviews. LLMs, such as ChatGPT, have demonstrated applicability across a range of academic writing tasks, including manuscript preparation, drafting discharge summaries, and generating educational content [18]. However, concerns regarding accuracy are substantial: current LLMs cannot access real-time literature, rely on training data with defined cutoff dates, and require the manual input of verified clinical information to maintain factual reliability [18]. Cheungpasitporn et al. identified that, in critical care nephrology, generative AI offers promising applications in literature review and academic writing, while acknowledging that ethical implications, data privacy concerns, and the necessity for sustained human oversight remain critical barriers to unmediated deployment [19].

Machine learning approaches, including text mining and citation screening automation, are actively being explored as tools to accelerate evidence synthesis and guideline development [2]. These developments indicate that while future narrative reviews may benefit from computational tools that ease the task of literature identification, these tools will not replace the essential expert interpretive function that defines and remains the irreplaceable strength of this format.

## Conclusions

Narrative reviews occupy an indispensable position in the contemporary medical literature. They condense heterogeneous bodies of evidence into clinically accessible syntheses, inform practice in areas where structured evidence is limited, and serve as durable educational vehicles for medical professionals. Their main problems are selection bias, poor reproducibility, and poor reporting standards. These can be fixed by using structured quality tools like SANRA and by editors checking search transparency and scientific reasoning more thoroughly. As the evidence base expands and new technologies reshape knowledge synthesis, the narrative reviews will continue to evolve. A thoughtful integration of transparency standards, domain expertise, and judiciously applied computational tools will determine whether narrative reviews continue to fulfill their potential as trusted, timely, and authoritative guides to the medical evidence landscape.
